# How does machine learning bias in prediction of behavioral phenotypes relate to individual characteristics?

**DOI:** 10.21203/rs.3.rs-10604176/v1

**Published:** 2026-09-29

**Authors:** Jianxiao Wu, Jingwei Li, Simon Eickhoff, B.T. Yeo, Sarah Genon

**Affiliations:** Research Center Jülich; Research Center Jülich; Research Center Juelich; National University of Singapore; Research Centre Jülich

**Keywords:** developmental neuroscience, machine learning, model bias, resting-state functional connectivity, socioeconomic status

## Abstract

Brain-based prediction of behavioral phenotypes is promising for precision medicine. However, predictive models in the field have commonly shown limited accuracy and individual bias. For instance, participants of ethnic/racial minority (e.g., African Americans) are more likely to be misclassified or show larger prediction errors, compared to white American participants. Addressing these limitations requires a better understanding of the sociodemographic and scan-related factors related to prediction biases. Here, we investigated the association between the prediction errors of brain-based models predicting over 100 behavioral measures and eight variables capturing sociodemographic and scan-related individual characteristics. These associations were examined in two developmental and one young adult cohorts (sample size = 4278, 432, and 784 respectively). We found that socioaffective and mental health phenotypes in developmental cohorts were particularly susceptible to bias associated with individual characteristics. In particular, head motion, head morphology, and sex/gender are consistently related to the machine learning biases in multiple behavioral measures. Our work highlights the multifactorial nature of the prediction errors generated by the brain-based predictive models. Overall, our results call for greater attention to model bias in developmental populations, particularly in the context of AI-based approaches for psychiatric risk prediction and early diagnosis.

## 1. Introduction

The application of machine learning in neuroscience has been widely used to distinguish patients with neurocognitive disorders from healthy individuals, to identify putative disease subtypes, and to predict treatment outcomes (1–4). More recently, this line of research expanded to the prediction of individual behavioral phenotypes from brain imaging data (5–9), often with the implicit expectation that such approaches may yield clinically useful, objective biomarkers for psychiatry and neurology (10, 11). For instance, many recent studies have reported discoveries of brain markers for general or specific cognitive abilities (6, 9, 12–14). However, despite substantial methodological development, reported predictive performance has frequently been modest, raising questions about their robustness.

In a standard predictive modeling framework, a model is trained on a set of individuals to learn associations between brain-derived features and a behavioral phenotype, and its performance is subsequently evaluated on independent, unseen data. With such a framework, a crucial goal of predictive modelling, for the deployment in precision medicine, is to ensure that the predictive models perform consistently across individuals with different social backgrounds or brain morphology. However, such bias-free performance is still lacking in current predictive models (8, 15). As a more specific example, we have previously shown that standard predictive models perform worse for African Americans than for white Americans (16). Furthermore, we demonstrated that this prediction bias across racial/ethnic groups cannot be fully addressed by improving model design, as certain inevitable biases may originate from the racial bias in neuroimaging data collection (17). Additional evidence highlighted that predictive models are systematically biased against specific individuals, whose phenotype-sociodemographic relationships differ from the sample stereotypes (15).

Overall, the causes of predictive model bias across individuals appear to be reflected by individual characteristics or traits that relate to brain-behavior relationships differently across individuals. However, it is unclear what exact factors underlie predictive model biases. A comprehensive investigation into the available individual characteristics in open datasets and their relationship with predictive model bias is therefore warranted. Importantly, moving beyond coarse sociodemographic stratifications such as ethnicity or age, we hypothesize that scan-related and neurobiological confounds may constitute key intermediaries of apparent demographic bias in brain-based predictive models. Accordingly, in addition to sociodemographic variables, we also examined indices of brain morphology and in-scanner head motion, both of which have been repeatedly shown to influence the validity and reliability of neuroimaging studies (18–21).

In this study, we systematically examined the relationships between individual characteristics and prediction bias across a broad range of behavioral phenotypes (see overview in Fig. 1). We leveraged three large-scale open neuroimaging datasets comprising high-quality brain imaging data and over 100 behavioral measures, with a focus on developmental and young adult populations. Behavioral outcomes were selected to span multiple domains, thereby capturing the breadth of typical applications in predictive modeling and enabling an assessment of whether associations between individual characteristics and prediction bias vary across behavioral domains. Finally, we quantified the unique contribution of each individual characteristic within a multivariate framework, identifying the most consistent drivers of prediction bias across models. Together, these analyses provide a more detailed characterization of the factors contributing to heterogeneity in brain-based prediction performance, and help delineate priorities for further work on the mechanisms underlying predictive bias.

## 2. Methods

We analyzed three publicly available neuroimaging datasets from developmental and young adult populations that are widely used in brain-based behavioral prediction: the Adolescent Brain Cognitive Development cohort (ABCD (Pre)-Adolescence; age: 9–11), the Human Connectome Project – Development (HCP Development; age: 5–21), and the Human Connectome Project – Young Adults (HCP Young Adult; age: 22–37). For each dataset, we derived resting-state functional connectivity features and selected between 22 and 51 behavioral measures spanning multiple domains. Predictive models were then built separately for each behavioral phenotype using cross-validation. Following standard practices in the field, the effects of common confounding variables were removed from the behavioral variables with linear regression. This was done during cross-validation, such that confound regressions were only estimated using the training subjects, in order to avoid data leakage.

### 2.1. Data and preprocessing

The ABCD release 5.0 included more than ten thousand pre-adolescents from 21 sites across the United States (22). We selected 35 behavioral measures encompassing the majority of available neurocognitive and mental health assessments (Table S1), consistent with our previous work (6, 16). After quality control and exclusion of participants with incomplete neuroimaging or behavioral data, 4,278 individuals were retained for analysis. For the Human Connectome Project (HCP) cohorts, we used data from the Lifespan HCP release 2.0 (23, 24) and the HCP-YA S1200 release (25). For behavioral data, we selected 22 cognition and emotion measures in HCP Development as well as 51 cognition, personality, and emotion measures in HCP Young Adult (see Table S2 and S3). Based on data availability, 432 HCP Development participants and 784 HCP Young Adult participants were retained for analysis.

For all datasets, we used neuroimaging data that had undergone ICA-based denoising and further applied nuisance regression to remove white matter and cerebrospinal fluid-related variance. Notably, preprocessing in all datasets involves adequate motion denoising. The preprocessed timeseries of each subject were concatenated across runs. Finally, functional connectivity matrices were computed across the 400-area Schaefer parcellation (23) and 54-region Melbourne subcortical parcellation (24) using Pearson’s correlation. Additional details on the data and preprocessing can be found in the Supplement.

### 2.2. Individual characteristics

We selected eight sociodemographic variables and scan-related variables to capture different dimensions of individual variability. First, five sociodemographic variables available in all datasets were selected including age, sex/gender, ethnicity/race, education, and household income. For the ABCD (Pre)-Adolescence dataset, only parental education was collected and was hence used instead of the participant education. In addition, inter-individual differences in brain morphology were indexed using intracranial volume and the Euler characteristic. The latter reflects global variability in cortical surface topology relative to the standard surface mesh. Finally, in-scanner head motion was quantified using framewise displacement during resting-state acquisition. Given that these variables are not independent, we characterized their interrelationships using appropriate pairwise association measures. Specifically, we used Pearson’s correlation for pairs of continuous variables, Cramer’s V for associations between categorical variables (see Supplementary Methods for details), and logistic regression for associations between mixed variable types.

### 2.3. Machine learning models

Machine learning models were built using functional connectivity features to predict each behavioral measure independently (Fig. 1A). Cross-validation schemes were adapted to the structure of each dataset to minimize potential sources of bias in model evaluation (see the Supplement for more details). Specifically, for the ABCD (Pre)-Adolescence and HCP Development datasets, site-based cross-validation was implemented to avoid involving site effects in model evaluation. For the HCP Young Adult dataset, care was taken to preserve family structures in the cross-validation scheme.

To remove confounding effects, common confounding variables were regressed from the behavioral measures using standard approach in the field (i.e. linear regression). These confounds include age, sex/gender, education, intracranial volume, head motion (using framewise displacement), and signal artefact (using DVARS).

The primary analyses focused on linear predictive models, as nonlinear models may amplify residual confounding when combined with linear confound regression procedures (26). Specifically, we used the linear support vector regression (SVR) algorithm for all datasets. For comparison, we additionally implemented the same prediction procedure using kernel ridge regression (KRR) with a nonlinear kernel. Prediction error was calculated as the absolute difference between the predicted and the original behavioral scores. For each behavioral measure and each participant, one average prediction error across cross-validation folds was computed.

### 2.4. Clustering behavioral measures

Given the large number of behavioral measures considered in this study, we grouped them into categories for downstream analyses. Clusters were defined based on similarity in prediction error profile across participants (Fig. 1B). Specifically, for each pair of behavioral measures, we computed the Pearson’s correlation between the prediction errors across individuals. We focused on pairs of behavioral measures that are sufficiently similar in terms of prediction error (with a medium correlation effect) by applying a threshold of R = 0.3. Finally, clusters of behavioral measures were formed by grouping these sufficiently similar behavioral measures.

Within each cluster, the absolute prediction errors of the behavioral measures were normalized to have the same scale. A “summary” prediction error was then computed for each cluster by averaging the normalized prediction errors of all measures within that cluster.

### 2.5. Associations between prediction errors and individual characteristics

To investigate how prediction model bias relates to individual characteristics, we examined associations between “summary” prediction errors for each behavioral cluster and the eight variables of individual characteristics (Fig. 1C). Given evidence of covariance among these variables, we assessed their joint and unique contributions using a multivariate general linear model (GLM) framework. First, we fitted a full model including all eight individual characteristics as predictors of the summary prediction errors. We then constructed a series of reduced models, each excluding one characteristic at a time (e.g., a model without Euler characteristic), to evaluate the relative contribution of each variable. The overall strength of association between prediction errors and individual characteristics was quantified using the pseudo R-squared of the full model. (see the Supplement for description).

The association with, or the importance of, each individual characteristic was investigated by two metrics comparing the full model and its corresponding reduced model. First, we estimated the importance of each individual characteristic by the partial R-squared (see the Supplement for description). The partial R-squared values can be interpreted as the percentage improvement an individual characteristic brings over the reduced model in which that characteristic was omitted. Second, we assessed the importance of individual characteristics based on the Bayesian Information Criteria (BIC), quantified by the difference of BIC values between the reduced and the full model. Because BIC explicitly penalizes model complexity, this metric provides a more conservative assessment of the contribution of each individual characteristic.

Finally, for the individual characteristics identified as important by the analyses above, we examined the direction of their associations with prediction errors. Associations involving continuous variables were assessed using both Pearson’s and Spearman’s correlations, whereas binary variables were evaluated using two-sample t-tests. To reduce heteroscedasticity and improve distributional properties, continuous variables were natural log-transformed prior to analysis.

## 3. Results

### 3.1. Prediction performance and clusters of behavioral measures

Overall, prediction accuracies were consistent with previous reports from our group and others (16, 27–30), with largely similar results observed for the two algorithms (SVR and KRR, Figure S1 and S2 respectively). Across datasets, cognitive measures were generally more accurately predicted than self-reported mental health phenotypes, replicating a well-established pattern in the brain-based prediction literature (6, 27, 31, 32).

To identify robust patterns across the 108 behavioral measures included in this study, we grouped behavioral measures according to the similarity of their prediction errors profile from SVR and KRR (Fig. 2 and S3 respectively). Clustering solutions were highly comparable across algorithms, with the exception of an additional cluster related to positive affect identified in the HCP Young Adult dataset using KRR. More broadly, prediction error clustering largely recapitulated the underlying organization of behavioral domains, suggesting that model errors exhibit structured relationships across phenotypically related measures.

### 3.2. Associations between scan-related factors and sociodemographic variables

Our investigation of the associations between individual characteristics demonstrated important covariance between variables. We observed correlations between scan-related and sociodemographic variables (Figure S4), but also among sociodemographic variables. Hence, as expected, the strongest association was between age and education in the HCP Development cohort, while sex/gender and head size were highly related in all cohorts. More notably, ethnicity/race showed consistent associations with multiple dimensions of inter-individual variability. These included expected relationships with socioeconomic indicators such as household income and education, but also associations with scan-related factors, including head motion and measures related to brain morphology. Additionally, in the HCP Development cohort, age was moderately correlated with Euler characteristics and head motion.

### 3.3. Prediction errors of most behaviors are associated with individual characteristics

We first quantified the extent to which individual characteristics explained variability in prediction errors using the full general linear model (Fig. 3). Across behavioral clusters, prediction errors were generally associated with individual characteristics, although effect sizes were modest. The strongest association was observed for emotion recognition measures in the HCP-D cohort, for which individual characteristics collectively explained more than 10% of the variance in prediction error.

To further disentangle the contribution of each factor, we estimated variable importance using both partial R-squared and Bayesian Information Criterion (BIC) (Fig. 4 and S5 for SVR and KRR results respectively). Higher values on both metrics indicate greater contribution to explaining prediction error variance, as reflected in the corresponding heatmaps. Overall, variables identified as important by BIC represented a more conservative subset of those highlighted by partial R-squared, consistent with the stronger penalization of model complexity in the BIC framework. Effects of individual characteristics on prediction errors were generally more pronounced in the ABCD (preadolescence) cohort compared to the other datasets.

Focusing on the convergence across different machine learning models and variable importance assessments, we identified nine consistent associations between prediction errors of behavioral clusters and variables of individual characteristics. Sex/gender was always associated with the prediction errors of cognition, behavioral problems (CBCL), and prodromal psychosis behaviors in the ABCD (Pre)-Adolescence cohort. Head motion showed consistent contributions to the prediction errors of behavioral problems (CBCL) and prodromal psychosis behaviors in the ABCD (Pre)-Adolescence cohort, as well as the emotion recognition measures in the HCP Young Adult cohort. Finally, the individual intracranial volume consistently contributed to the prediction errors of cognition and behavioral problems (CBCL) in the ABCD (Pre)-Adolescence cohort, as well as the emotion recognition measures in the HCP Development cohort.

### 3.4. Model biases were related to larger head motion and smaller head size

Focusing on the key individual characteristics identified in the multivariate analyses—sex/gender, head motion, and intracranial volume—we examined their univariate associations with the corresponding prediction errors (Table S4). Associations involving head motion and intracranial volume were assessed using both Pearson’s and Spearman’s correlations, whereas sex/gender effects were evaluated using two-sample t-tests.

Consistent patterns were observed across behavioral clusters for the same individual characteristics. Overall, higher prediction errors were associated with greater head motion, smaller intracranial volume, and male sex/gender. However, when restricting inference to rank-based associations (Spearman’s correlation), the effect of intracranial volume was primarily observed for behavioral problem (CBCL) measures in the ABCD (preadolescence) cohort.

## 4. Discussion

In this study, we investigated how prediction bias in brain-based behavioral modeling relates to individual characteristics across ten categories of behavioral phenotypes and for three young populations cohorts. Our findings indicate that prediction errors are not randomly distributed across individuals, but are systematically associated with sociodemographic and scan-related factors that themselves strongly covary. Importantly, these associations were most consistently observed for socio-affective/mental health phenotypes in developmental cohorts. This observation is particularly relevant given that such measures are among the most common targets for the development of neuroimaging-based biomarkers in psychiatry, yet also among the most difficult behavioral phenotypes to predict from brain data. Together, our results suggest that limited predictive performance in psychiatric phenotypes may partly reflect systematic prediction biases linked to individual characteristics, rather than only weak brain–behavior associations.

Across cohorts and behavioral domains, prediction bias was more strongly related to individual characteristics for socio-affective/mental health measures than for cognitive measures. Previous work has consistently shown that socio-affective and psychiatric phenotypes yield lower prediction accuracies in brain-based modeling studies, often attributed to factors such as lower test–retest reliability and the subjective nature of self-report assessments (8, 33, 34). Our findings extend this literature by suggesting that prediction biases associated with individual characteristics may constitute an additional and underappreciated source of performance limitation. Notably, these effects persisted despite standard confound regression procedures applied during model training, indicating that commonly used preprocessing and statistical correction strategies may be insufficient to fully mitigate systematic bias in predictive modeling pipelines. These observations have direct implications for translational neuroimaging and precision psychiatry, where predictive models are increasingly proposed for individualized risk stratification, prognosis, or early diagnosis.

Previously, we found that the brain-based predictive models of behavioral measures tend to perform worse in African American participants compared with white American participants (16). The present findings suggest that such racial/ethnic disparities in model performance may partly emerge from covariance between race/ethnicity and other dimensions of individual variability. Across all cohorts, race/ethnicity was associated not only with socioeconomic indicators, but also with scan-related factors such as head motion and measures related to brain morphology. These findings raise the possibility that demographic prediction bias may partly reflect systematic differences in data quality or neuroimaging-derived measures across groups. Consequently, balancing datasets based solely on racial/ethnic composition may not be sufficient to eliminate predictive bias, as shown in our previous study (16). Our results support the hypothesis in our previous study that the racial/ethnic bias may partly originate from the lack of efficacy in standard neuroimaging pipelines for the racial/ethnic minority groups. In light of such results, we encourage future studies to assess the effectiveness of preprocessing pipelines across racial/ethnic groups.

Relatedly, multivariate analyses indicated that head motion and intracranial volume accounted for a substantial proportion of the explained variance in prediction bias. This suggests that associations between prediction bias and sociodemographic variables may, in part, be mediated by systematic differences in scan quality and brain morphology across groups. The relationship between prediction bias and head motion likely reflects the well-established sensitivity of functional connectivity measures to motion-related artifacts (19, 35), particularly in developmental cohorts where motion is more variable (24). In contrast, the association with intracranial volume was more specific to developmental samples and may relate to variability in neurodevelopmental trajectories. Brain size deviations have previously been reported in neurodevelopmental conditions such as autism spectrum disorder and attention-deficit/hyperactivity disorder (ADHD) (36–38). In a healthy population, total brain volume was also related to self-reported ADHD assessment scores such as inattention (39). Disentangling confounding effects from biologically meaningful variability therefore remains a major challenge in developmental neuroimaging (40). Our results support the need for a deeper understanding of the interactions between head size and neurocognitive phenotypes.

Lastly, sex/gender contributed substantially to explaining prediction bias in the ABCD (preadolescence) cohort, with male participants generally showing larger prediction errors than female participants. Sex/gender differences in brain development are well documented, although their effects on functional connectivity organization remain incompletely understood and sometimes inconsistent across studies (41–44). Interestingly, previous work has suggested that brain structural measures may exhibit greater inter-individual variability in males compared with females (41, 45). One possible interpretation is that the higher prediction errors observed in male participants reflect analogous differences in variability within functional brain organization. This hypothesis should be investigated in future studies while specific attention should be paid to sex/gender-specific bias in development cohorts.

Several limitations should be considered when interpreting these findings. First, prediction errors in regression-based models are inherently influenced by regression dilution effects, whereby predictions tend to regress toward the population mean and errors become larger at more extreme behavioral values (46–48). In the present study, it is therefore difficult to fully disentangle the contribution of regression dilution from the contribution of individual characteristics, as both may jointly influence prediction error distributions. Second, although many associations were consistent across datasets and modeling approaches, effect sizes were generally modest. Importantly, because confounding variables were already regressed out during predictive modeling, the remaining associations between prediction bias and individual characteristics may also reflect residual effects persisting after standard confound correction procedures. From this perspective, our findings may indicate both the relative effectiveness and the limitations of commonly used preprocessing and deconfounding strategies.

Through a systematic investigation of variability in prediction bias across individuals, we demonstrate that machine learning bias in brain-based behavioral prediction reflects the combined influence of multiple interrelated sociodemographic and scan-related factors. In particular, head motion, intracranial volume, and sex/gender emerged as consistent contributors to prediction bias, especially for socioaffective and mental health phenotypes that are central to current efforts in psychiatric biomarker development. For future studies, we recommend explicit consideration of individual characteristics with potential confounding effects, such as examining sex/gender-specific outcomes, reporting head motion statistics across sociodemographic groups, and analyzing interaction or mediation effects of brain morphology on neurocognitive measures of interest. More broadly our findings call for greater attention to model bias in developmental populations and for AI-based applications in psychiatric disorder diagnosis.

## Supplementary Material

Supplementary Files

This is a list of supplementary files associated with this preprint. Click to download.

- PredErrsupp20260429.pdf

## Figures and Tables

**Figure 1:**
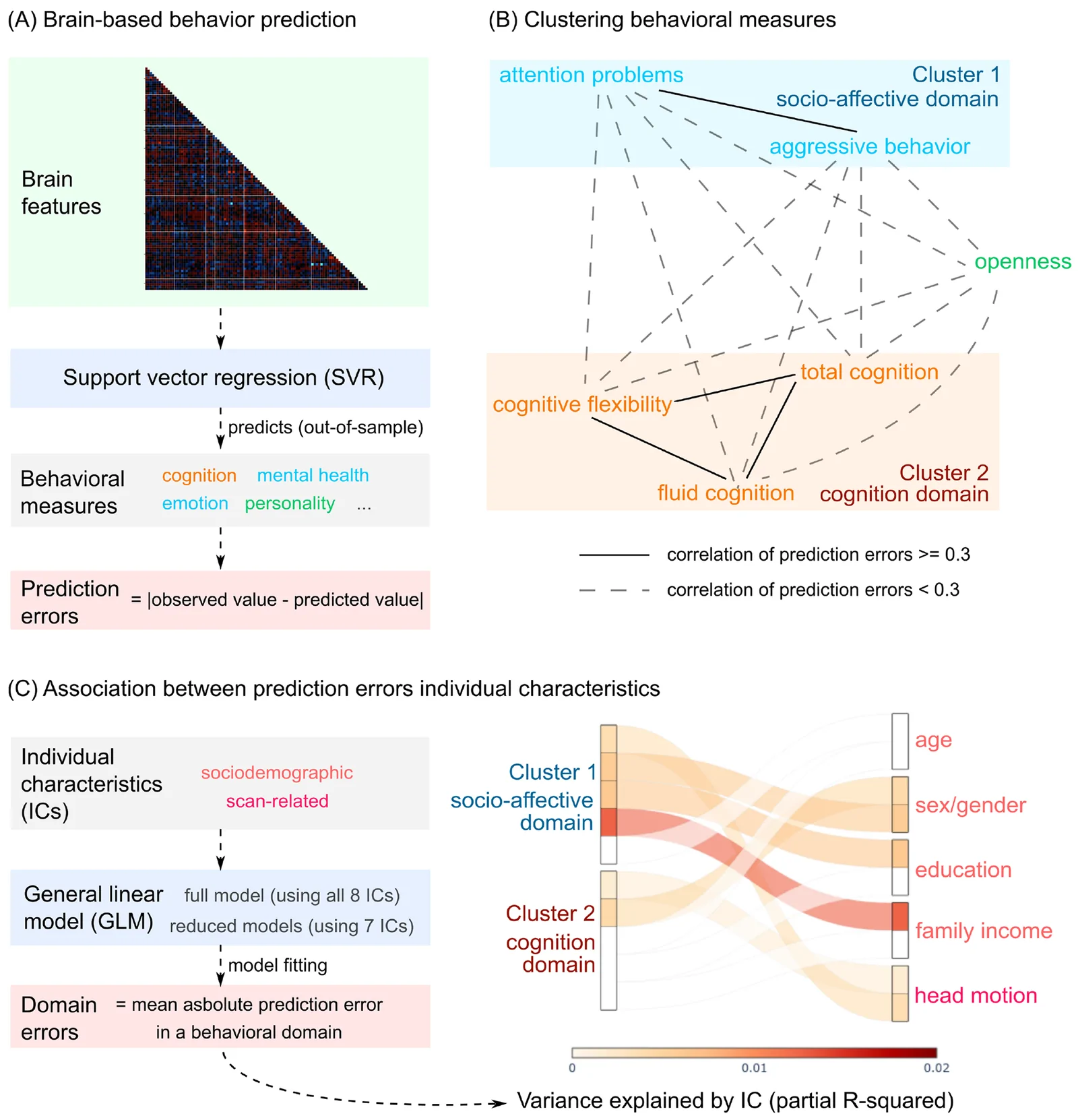
Overview of study design. First, we implemented standard brain-based behavior prediction to predict a range of behavioral measures. We then clustered the behavioral measures based on their prediction error profiles. Finally, we characterized the association between the prediction errors of the behavioral clusters and variables of individual characteristics, using a multivariate general linear model framework.

**Figure 2:**
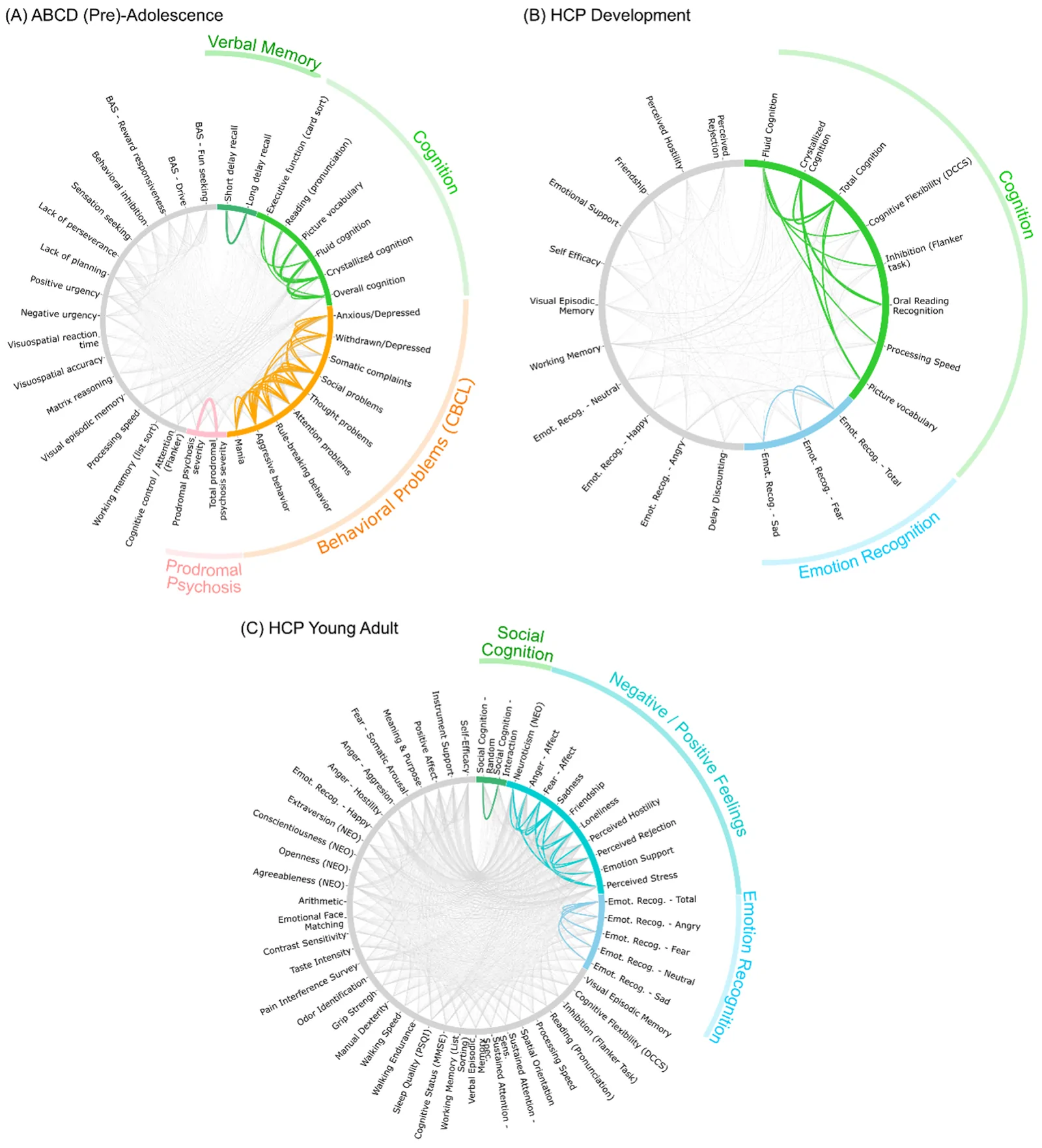
Clusters of behavioral measures in (A) ABCD (Pre)-Adolescence, (B) HCP Development, and (C) HCP Young Adult datasets based on prediction errors from support vector regression (SVR).

**Figure 3:**
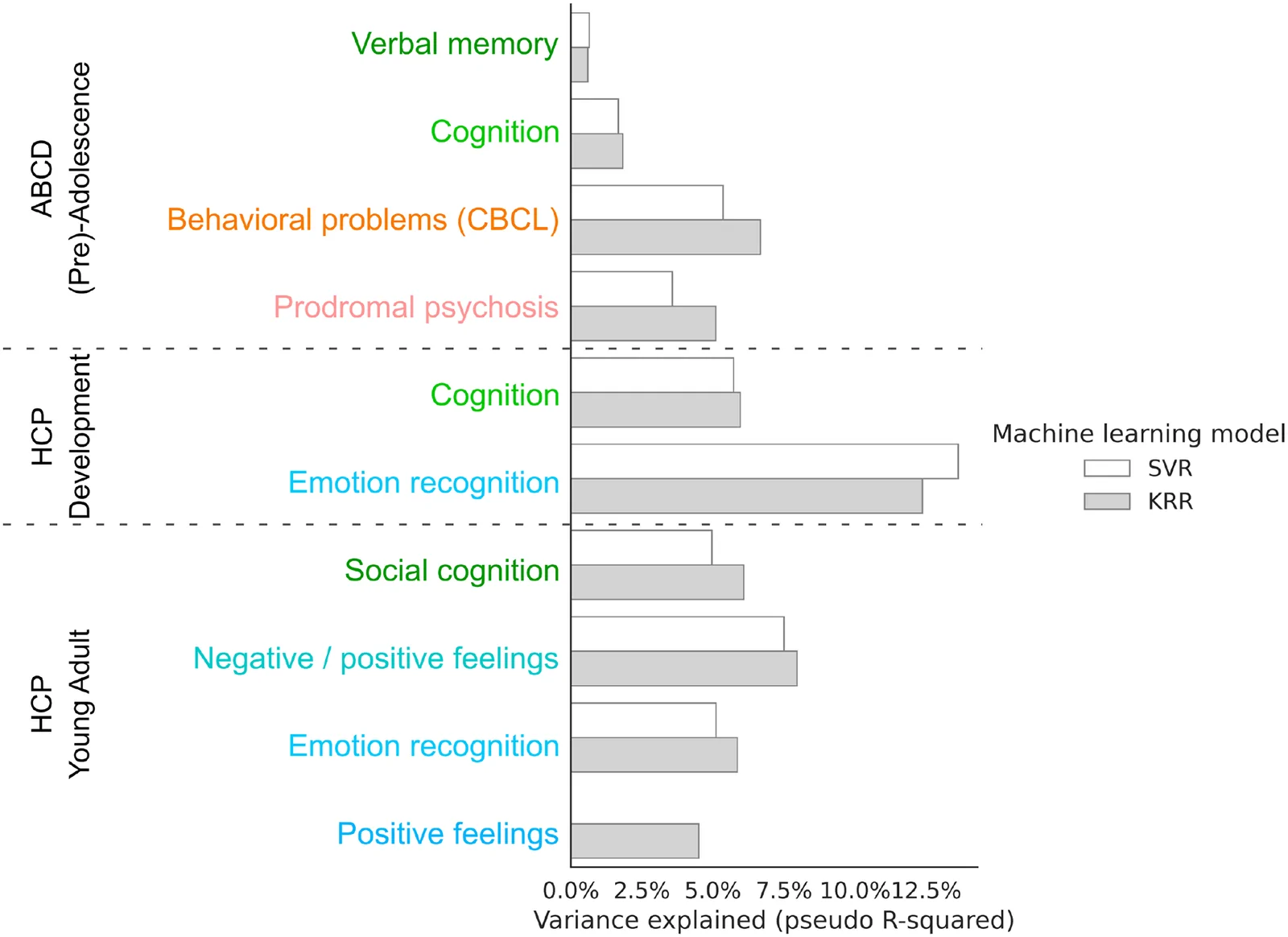
Variance in prediction errors explained by full general linear models using all eight individual characteristics, represented by pseudo R-squared.

**Figure 4:**
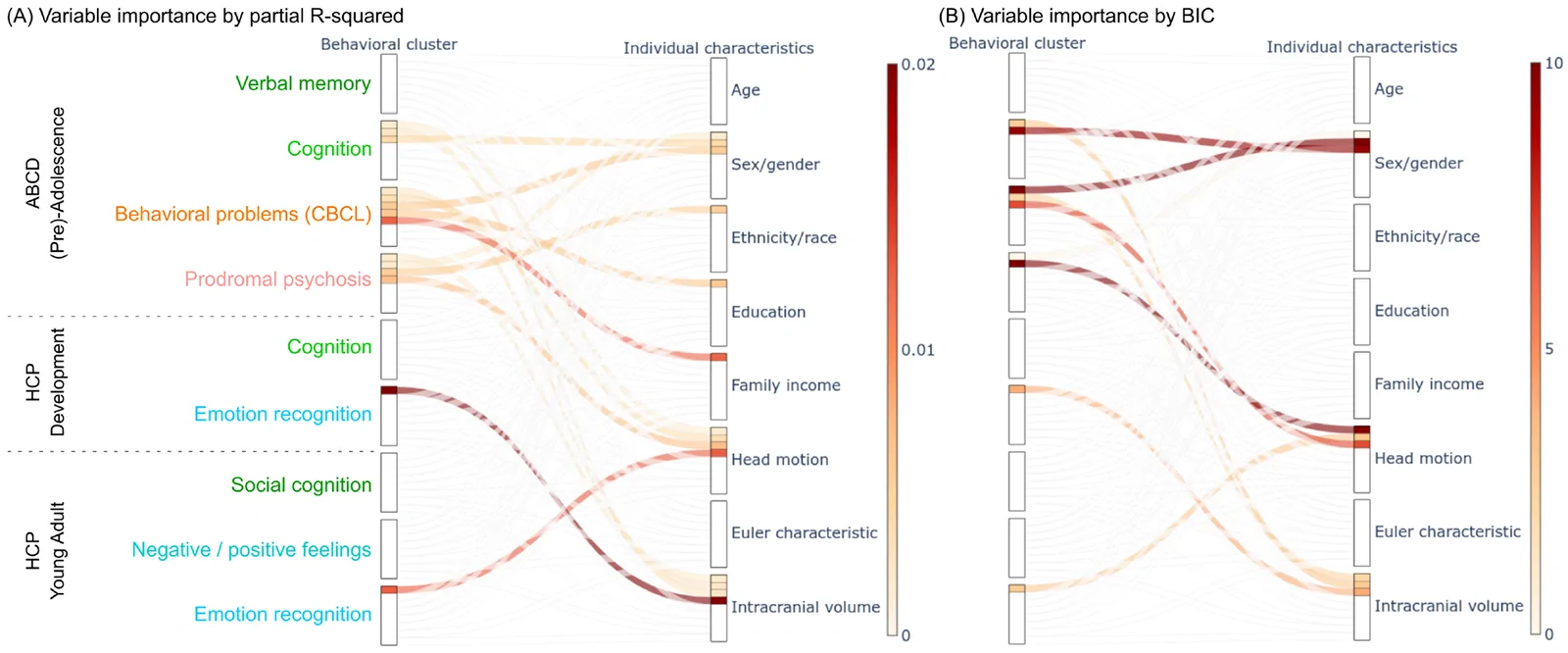
Importance of individual characteristics in explaining prediction errors across behavioral clusters based on (A) partial R-squared and (B) Bayesian Information Criterion (BIC). Results are shown for prediction errors derived from the SVR models. Warmer colors (towards red) indicate greater importance of a given characteristic, whereas white indicates no detectable contribution.

## Data Availability

All data were managed via version-controlled DataLad datasets that are either publicly available, or were provided by an institutional data management system when public sharing was prevented by the terms of the respective data usage agreements. The HCP Young Adult imaging data were accessed via the public DataLad dataset provided at https://github.com/datalad-datasets/human-connectome-project-openaccess (2e2a8a70-3eaa-11ea-a9a5-b4969157768c@a33e528) which interfaces the HCP Open Access dataset (https://registry.opendata.aws/hcp-openaccess) on AWS S3. The unrestricted and restricted phenotype data were downloaded from the ConnectomeDB (https://db.humanconnectome.org) after accepting the Open Access Data User Terms and Restricted Access Data Use Terms respectively. The HCP Development and ABCD (Pre)-Adolescence imaging and phenotype data were downloaded from the NIMH Data Archive (NDA; https://nda.nih.gov), after applying for the Data Use Certification. The associated study ID is 3290 (https://dx.doi.org/10.15154/8e8k-jf34). All code for analyses in this study are publicly available at https://github.com/jingwei-li/PredErr_vs_multifactors. The code for brain feature extraction from HCP data are available at https://github.com/jadecci/mpp.
